# Use of Hypochondriacs

**Published:** 1876-10

**Authors:** Ben. H. Pratt


### THE BISTOURY.

Vol. XII. ELMIRA, N. Y., OCTOBER, 1876. No. 3.

### Contributions.

### THE USE OF HYPOCHONDRIACS.

BEN. H. PRATT.

The man who does the most good without knowing it, is the hypochondriac. True, he isn't jolly as a companion—one don't go into hysterics of laughter in his presence—one can't say of him that he's an entertaining conversationalist — humor doesn't bubble up spontaneously from his veins, nor do peals of wit drop in glittering globules from his glibly trilling tongue. One does not become saturated with mirth, nor catch a spell of pleasantry from being in his presence an hour or two, nor does a thoughtful contemplation of his face tend to thrill one with a sense of ecstasy. The catalogue of a hypochondriac's woes, as narrated by himself, a recital of his ills, a list of his maladies, a detailed programme of the course of treatment to which he has been subjected, a schedule of the doctors he has summoned upon his case, the varied diagnoses of each, long diatribes against the medical skill of the age, a running treatise upon the humbugging of medical practice, a general summing up to the effect that all the schools of the so-called healing art are founded upon theories based on guesses—all this isn't calculated to make the hypochondriac's visitor thoroughly and entirely blissful. It isn't a hypochondriac's forte to directly imbue another with any great amount of genuine jocularity. And yet he is the indirect cause of more real fun, the author of more healthful humor, the most fruitful source of rib-racking laughter, of all the would-be wits of the region in which he dwells.

The man who thinks he is sick, but isn't—who, by some quirk in his mental make-up, imagines himself the victim of some one or a score of insidious maladies, no one of which human skill can reach, and which are so subtle in their origin and development as to be far beyond the keenest ken of Æsculapian research—who prides himself upon the supposition that he is the indwelling temple and sole proprietor of a whole host of diseases whereof no man knoweth—that he bears about within himself afflictions which are as truly hidden and incomprehensible as the *arcanæ* of the gods —who has long since ceased to take any interest in anything beyond the pale of his own abnormality—who, like the alchemist, is continually searching for something which he is confident can never be found—who bends all his powers of thought and imagination into the one channel of self-introspection, or mental peering within himself—who is constantly on the watch after new symptoms, and deems that day a peculiarly unhappy one which does not reveal some new phenomena of ache, pain, distress or other novelty in the development of one or more of his pet affections—he is indeed a good subject for merciless discussion, a fair mark for the gibes of a widely extended neighborhood, and a butt among all who have an acquaintance with the case, however limited.

Himself unconscious of his wide spread popularity as a subject of conversation, sitting or lounging alone, with no hint as to the frequency with which his name is mentioned in the social circle from which he is a self-created exile; he never even dreams how much he contributes toward the rare sport of his circle of acquaintances. He is innocent of his negative qualities as a fun-maker, and hasn't the remotest idea that he is the source of perpetual joy, a perennial rivulet of humor, a well-spring of merriment—one who would be more regretfully missed from the community than the brightest wit or drollest clown it affords.

The chronic hypo is so supremely miserable that the gloomiest of ordinary mortals seems a happy man in the comparison. Hence the latter is often urged to visit the former, and always with good results. It makes people happy to know that there are folks ten-fold more miserable than themselves. The average gloomy man, after a conference with a confirmed hypo, comes forth lightened of half his load of dismal thoughts, and is almost tempted to believe that he really has much to be thankful for. Being fearful lest he may, by fostering his leaven of gloom, become such an one as he from whom he has just departed, he uses his best efforts to abandon his darker broodings, and endeavors to glean what of cheer he may from mingling with those who possess an ordinary share of good nature, and a normal flow of good spirits.

In the social circle which chances to embrace a hypochondriac within its borders, there is never lacking an abundant supply of material for talk and fun, since, when all other sources fail, the latest ailment of our hypochondriacal friend, affords an ample and a pleasing field for discussion, which invariably runs into the serio-comic or broadly ludicrous. The last whim of the sick man may only be the possession of a tape-worm, or a stomach pet in the form of a lively lizard, or a new fangled hepatic parasite, the genus and species of which are not yet fully determined by the naturalist; but even such trifling matters as these are readily worked up into intensely interesting subjects when handled by skillful conversationalists. The imagination of a coterie of parlor gabblers has such a fine opportunity to run riot with the poor devil's cherished collection of zoological and parasitical specimens—can conjure up so many varied antics which these little creatures might cut up inside our friend's anatomy, all alone in the dark there—can so curiously wonder whether a pet stomach lizard would prefer a bolus of milk toast or a fresh raw oyster for a couch, after his romp of the day was over—can quaintly conjecture with what eagerness, mayhap, the pale little fellow sits up on his hind legs, propped by his tail, at the esophagal gate of the stomach, watching for a fresh tit-bit for his breakfast, and holding out both sprawling paws to catch the morsel before it becomes submerged in the rather stale invoices of the previous day—can imagine how, after our hypoic hero has fasted a day or so, the little reptile might make prodigious efforts to scramble up the gullet in his impatience for a fresh morsel, and after using his toes, tail and spines vigorously for an hour or so, succeed in getting stuck midway, like a chimney sweep, one size too big for the flue—can dwell upon the peculiar sensation which such a wiggling around in that region might produce upon the highly sensitive organism of our hypochondriac friend, and almost shudder at thinking what a chill the lizard must experience should ice-water be resorted to in a case of this sort—in a word, there's no limit to the delightful variety of interesting situations which can be invoked to give spice and vigor to a case like this, where a guileless lizard and a nervous hypo are having duets of this sort. Should, however, the theme grow tiresome, the introduction of the tape-worm upon the tapis—or rather within the stomach—would open up another and more prolific source of amusement, besides proving a very profitable intellectual exercise, partaking of the nature of those puzzles in which we endeavor to ascertain into how many shapes a set of serpentine blocks may be arranged. Another very entertaining exercise might be instituted by theorizing, as scientifically as one may choose, as to whether the acephalous sub-divisions of this specimen of tænia ever assumed cephalous characteristics, or whether the dismembered parasite may not again assume unity. Surely such contemplations, together with such others as the ingenuity of the company might suggest, could be made very cheerful, at least to those who were not personally interested in lizards, tape-worms and the like, however queer he might take it who furnishes lodgings and board within himself for the pet creatures under discussion.

Again, supposing that a party of our hypochondriac's friends should visit the Centennial, will he not be frequently called to mind when that department is visited which embraces the exhibits of drugs and medicines? How satisfyingly would the monuments of epsom and glauber salts be contemplated, inasmuch as when the party returns home it may confidently assure their friend, who may have grown anxious upon this point, that the supply of these very active ingredients of our pharmacopia is not at all likely to fall short, even though the demand should be doubled. And then the pills! those vast pyramidal mounds of all sorts and kinds of pills! Pills in vials and jars and casks and even in hogsheads; pills little and pills big; pills sugar-coated and pills bare; pills white and pills brown; pills to suit the most whimsical swallower and the most fastidious taster and the most capacious ailer; arranged with so much taste and vying in beauty with the less potent exhibits of the confectioners. With the feminine enthusiast of the centennial we may well exclaim, "how beautiful are pills!" and add, how searching, piercing even to the dividing asunder of the soul and body, of the joints and marrow! What a tibute to the skill and the ingenuity and the taste and the genius of the age is this magnificent and munificent collection of pills at the Centennial! And, moreover, what a boon to mankind, and what a consolation to the hypochondriac, and how inspiring! How eloquent one might become in narrating of these to our hypochondriac friend at home upon our first visit to his chamber after a trip to the American Mecca. And how he would rejoice in the thought that, however delightfully complicated his maladies may yet become, even beyond his most sanguine expectations, there are still hills of pills and squills, and mills to fashion more, so long as ills abound, and doctors' bills are popular. Moreover, behold what columns of nitrates! what pillars of carbonates! what obelisks of phosphates! what placebos for magnates in the invalid world! how one might fairly ravish our hypo friend's ears with our medicated eloquence, and stuff him chock full of melancholy joy at the prospect of indefinitely prolonging the sweets of invalidity.

Thus, as the misfortunes of one man invariably redound to the good fortune of other men, and the sorrow of one to the joy of another, so does the melancholy of the hypochondriac prove a source of unlimited diversion to his fellows. And hence, he who would uncompromisingly and unconditionally class the hypo among those who bring no measure of joy into the world, but only cast darkling shadows to begloom the pathway of those who might be happy but for him, most surely errs in his estimate of the fitness of things and in determining the hypochondriac's place in the social regime. In the grand economy of the theologue, there must be devils, that seraphs may the more abundantly shine forth and not appear as namby-pamby perfections; in the every-day economy of well-living, there is work, that play may the better be enjoyed; thorny stalks bear the fragrance and beauty which roses bring; pain abounds, lest we forget the joys of health in the perpetual possession of those joys; there are thieves and robbers and murderers and evil doers of every grade, to the end that honesty and uprightness may appear the brighter by the contrast; and therefore are these hypochondriacs, whose melancholy and gloom and bitter broodings over imaginary ills may bring out all the more splendidly the roseate phase of a healthful and a normally social existence. Men are happy or miserable only by comparison. When, therefore, such glorious contrasts as the hypochondriac offers us are afforded for our more perfect happiness; and whereas he seems to enjoy misery, in his way, as thoroughly as others enjoy happiness, in their way, we certainly ought not to despise him, and he scarcely deserves our pity, nor need he ever know how much he contributes to our enjoyment.

A famine is said to prevail in northern provinces of China.

